# Supplementary material for: Venomix: a simple bioinformatic pipeline for identifying and characterizing toxin gene candidates from transcriptomic data
Source: PeerJ. 2018 Jul 31;6:e5361. doi: 10.7717/peerj.5361 (PMC6074769; doi:10.7717/peerj.5361)
Supplement: Supplemental Information 3 [file peerj-06-5361-s003.gz › FinalOutput_E-6/Glutaminyl-peptide_cyclotransferase_1/finaltree.pdf]

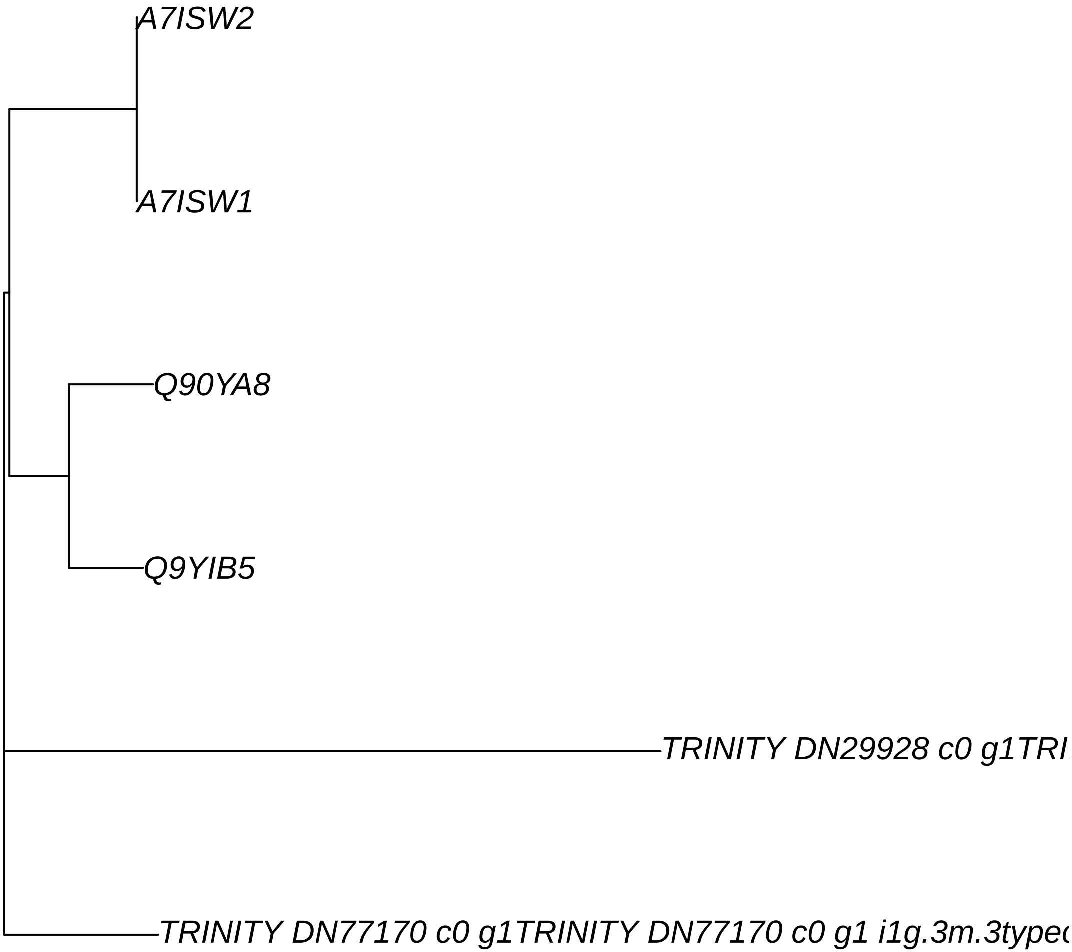
A7ISW2
A7ISW1
Q90YA8
Q9YIB5
TRINITY DN29928 c0 g1TRI
TRINITY DN77170 c0 g1TRINITY DN77170 c0 g1 i1g.3m.3typed
